# SUPPORTING NURSING HOME MANAGERS TO ACT ON PERFORMANCE FEEDBACK DATA: A PROVINCEWIDE IMPLEMENTATION

**DOI:** 10.1093/geroni/igac059.1520

**Published:** 2022-12-20

**Authors:** Carole Estabrooks, Seyedehtanaz Saeidzadeh, Stirling Bryan, Peter Norton, Liane Ginsburg, Matthias Hoben, Don McLeod

**Affiliations:** University of Alberta, Edmonton, Alberta, Canada; University of Alberta, Edmonton, Alberta, Canada; Michael Smith Health Research, Vancouver, British Columbia, Canada; University of Calgary, Calgary, Alberta, Canada; York University, Toronto, Ontario, Canada; University of Alberta, Edmonton, Alberta, Canada; Translating Research in Elder Care, Edmonton, Alberta, Canada

## Abstract

INFORM (Improving Nursing home care through Feedback on perfoRMance data) is a tested research intervention targeting care managers that demonstrated positive two year follow up results and has subsequently been shaped into an operationally acceptable “implementation package”. This package or innovation is being scaled up in one Canadian province’s total Long-Term-Care (LTC) home population with in depth process evaluation during the first cohort of LTC homes. This evaluation will, among other things, assess sector needs for adaptation (vs fidelity). At its core INFORM is designed to address managers’ learning needs with respect to using data to make positive change in a continuous learning loop. We will discuss the transformation of a research intervention to a sector innovation and report on interim process evaluation results.

